# The Genetic Requirements for Pentose Fermentation in Budding Yeast

**DOI:** 10.1534/g3.117.039610

**Published:** 2017-04-10

**Authors:** Karin Mittelman, Naama Barkai

**Affiliations:** Department of Molecular Genetics, Weizmann Institute of Science, Rehovot 76100, Israel

**Keywords:** fermentation, overflow-metabolism, *Saccharomyces cerevisiae*, xylulose, Mutant screen report

## Abstract

Cells grow on a wide range of carbon sources by regulating substrate flow through the metabolic network. Incoming sugar, for example, can be fermented or respired, depending on the carbon identity, cell type, or growth conditions. Despite this genetically-encoded flexibility of carbon metabolism, attempts to exogenously manipulate central carbon flux by rational design have proven difficult, suggesting a robust network structure. To examine this robustness, we characterized the ethanol yield of 411 regulatory and metabolic mutants in budding yeast. The mutants showed little variation in ethanol productivity when grown on glucose or galactose, yet diversity was revealed during growth on xylulose, a rare pentose not widely available in nature. While producing ethanol at high yield, cells grown on xylulose produced ethanol at high yields, yet induced expression of respiratory genes, and were dependent on them. Analysis of mutants that affected ethanol productivity suggested that xylulose fermentation results from metabolic overflow, whereby the flux through glycolysis is higher than the maximal flux that can enter respiration. We suggest that this overflow results from a suboptimal regulatory adjustment of the cells to this unfamiliar carbon source.

The budding yeast *Saccharomyces cerevisiae* efficiently adjusts its central metabolism when presented with different nutrients in its environment. In particular, cells can regulate the distribution of carbon flux between fermentation and respiration, depending on carbon quality and availability. Thus, sugars that enter the cell are converted into pyruvate, which can be either directed toward mitochondrial respiration or fermented into ethanol (Figure 1A). The fate of pyruvate at this branch point is determined by the abundance and activity of different metabolic enzymes which, in turn, are subject to extensive transcriptional and post-transcriptional regulation.

**Figure 1:**
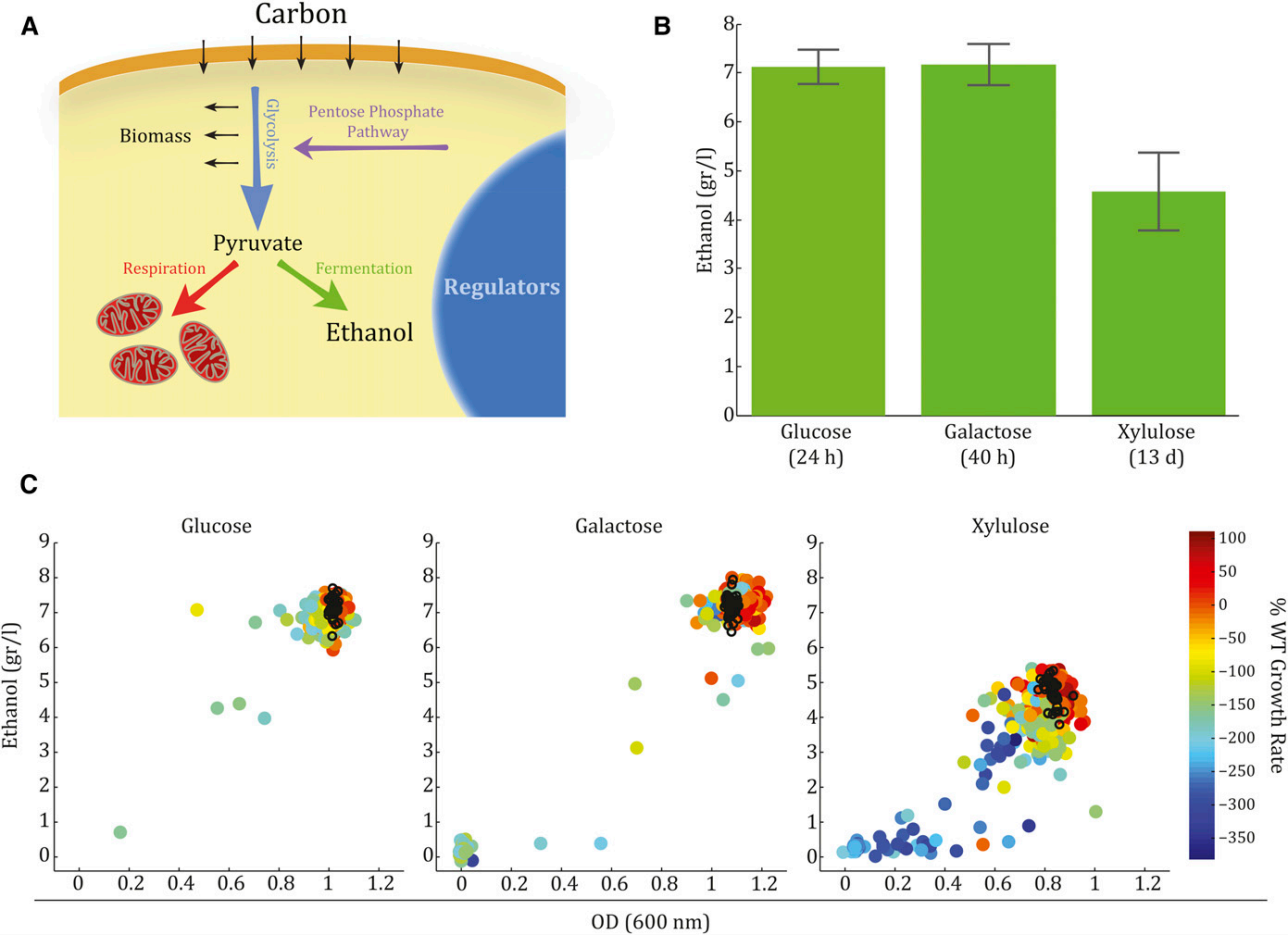
Ethanol production on xylulose differs from glucose and galactose in yield and variability. (A) A schematic overview of carbon metabolism pathways in yeast. Shown are the major metabolic pathways discussed, which include the genes included in our screen. (B) Wild-type (WT) cells produce different amounts of ethanol from different carbon sources. Shown are the amounts of ethanol produced by WT yeast grown on synthetic complete media with each of the three carbon sources used in the screen. Cells were grown until all of the sugar in the media was consumed (24 hr on glucose, 40 hr on galactose, and 13 d on xylulose) and the amount of ethanol produced was measured by high-performance liquid chromatography analysis. (C) Variability of ethanol production differs dependent on carbon source. The amount of ethanol produced by each of the deletion strains during growth on each of the carbon sources examined is shown *vs.* their final OD at stationary phase. The color code depicts the percentage of growth rate of the WT strain, as determined by a sensitive competition assay.

The flexibility of the metabolic network is exemplified by the metabolism of glucose and galactose, two structurally similar hexoses. Glucose is efficiently fermented into ethanol with only little oxygen consumption, while galactose flux is distributed between fermentation and respiration (Velagapudi *et al.* 2007). Indeed, the transcription program activated by each of these sugars is quite different; in particular, glucose triggers a large-scale transcriptional response called glucose repression, characterized by the downregulation of genes required for respiration or for metabolism of alternative carbon sources. This repression is largely alleviated in galactose-growing cells. In particular, galactose induces several of the glucose-repressed genes for growth (Johnston 1987; Gancedo 2008; Zaman *et al.* 2008).

Budding yeast has been extensively exploited in the bioethanol industry not only because of its efficient fermentation capacity, but also due to its tolerance for high ethanol concentrations and other stresses associated with mass fermentation (Olsson and Hahn-Hägerdal 1993; Olsson and Nielsen 2000; van Zyl *et al.* 2007). A potential feedstock for bioethanol production is lignocellulose, a polysaccharide found in agricultural crop waste, wood residues, and other industrial byproducts. Lignocellulosic biomass consists of ∼40% hexoses (mostly glucose) and 20% pentoses (mostly xylose). While budding yeast is highly efficient in fermenting the hexose fraction it does not metabolize xylose, largely reducing the potential ethanol yield from lignocellulose fermentation (Aristidou and Penttilä 2000; Matsushika *et al.* 2009). Attempts to engineer xylose-fermenting *S. cerevisiae* strains face multiple challenges, including the transport of xylose into the cell, isomerization of xylose into the metabolic intermediate xylulose (Hahn-Hägerdal *et al.* 2007; Matsushika *et al.* 2009; Laluce *et al.* 2012), and finally direction of xylulose metabolism towards fermentation, rather than respiration. While many studies have shown that different mutations can improve xylose fermentation by yeast (Kim *et al.* 2013; Sato *et al.* 2016; Bamba *et al.* 2016; dos Santos *et al.* 2016), overcoming the challenge of directing xylulose toward fermentation has received little attention, and requires better understanding of the genes and processes that control central carbon flux.

Despite being rare in the natural environment, budding yeast can grow on xylulose as a sole carbon source, enabling a direct study of the means by which cells metabolize this intermediate (Moses and Ferrier 1962; Gong *et al.* 1981; Chiang *et al.* 1981). Xylulose is transported into the cells via the hexose transporter family, it is then phosphorylated and enters central metabolism through the pentose phosphate pathway (PPP) (Hohenschuh *et al.* 2015). In this study, we systematically screened for metabolic genes that contribute to xylulose fermentation. We find that although xylulose is fermented at considerable yields, cells grown on xylulose activated the transcription program characteristic of respiring cells, and further relied on respiratory genes for growth. Analysis of mutants whose ethanol productivity was diverse revealed that mutants with similar fermentation capacities showed contrasting gene expression programs, and little correlation was found between their fermentation capacity and expression of respiratory genes. Rather, fermentation capacity appeared to correlate with growth rate. The model of overflow metabolism, which suggests that cells direct carbon toward fermentation only after exhausting their respiration capacity, can account for this growth pattern. Based on these results, we suggest that xylulose fermentation is governed by metabolic overflow, likely resulting from a suboptimal regulatory response to this rare carbon source, leading to a limiting respiration capacity.

## Materials and Methods

### Yeast strains and media

The 411 strains included in our screen were chosen following extensive literature research (Johnston 1987; Olsson and Hahn-Hägerdal 1993; Schüller 2003; Wang *et al.* 2004; Santangelo 2006; Gancedo 2008; Zaman *et al.* 2008, 2009) and include strains deleted for all metabolic enzymes that take part in central carbon metabolism, as well as the regulatory factors that affect these reactions, either directly or indirectly. These genes include sugar transporters, enzymes of glycolysis, gluconeogenesis, the PPP, fermentation, the TCA cycle, and the respiratory chain. In addition, genes required for the catabolism of specific carbon sources were screened: galactose, sucrose, fructose, mannose, xylose, arabinose, and glycerol. Genes of regulatory networks known to transcriptionally regulate carbon metabolism were included as well: the Ras/PKA network, the Snf1 network, and genes known to specifically regulate the above-mentioned pathways, at both the mRNA and protein levels. BY4741 *MATa his3*-Δ*1 leu2*-Δ*0 lys2*Δ*0 met15*-Δ*0 ura3*-Δ*0* cells were used as haploid wild-type. Strains deleted for the nonessential genes chosen were taken from the Yeast Knockout deletion collection (BY4741 genetic background). BY4743 *MATa/*α *his3*Δ*1/his3*Δ*1 leu2*Δ*0/leu2*Δ*0 LYS2/lys2*Δ*0 met15*Δ*0/MET15 ura3*Δ*0/ura3*Δ*0* cells were used as diploid wild-type. Heterozygotes for deletions of genes were acquired from the Yeast Magic Marker Collection (BY4743 genetic background).

Deletion strains created for validation experiments were derived from BY4741 using the LiAc/SS DNA/PEG method described by Gietz *et al.* (1995). In each strain, the gene deleted was replaced with the kanMX cassette (*gene*Δ::*KANMX*) using UPTAG and DNTAG primers as described in the Yeast Deletion Project (http://www-sequence.stanford.edu/group/yeast_deletion_project/usites.html). The deletion was then validated with primers A, B, and kanB as described in the project overview.

Strains were grown on standard SC media containing glucose, galactose, or xylulose (2%) as a carbon source.

### Xylulose production

A mixture of xylose and xylulose was enzymatically produced from D-xylose as previously described Gong *et al.* (1981). 350 g of D-xylose (Sigma X1500) were dissolved in 500 ml DDW and incubated with 20 g glucose isomerase (Sigma G4166) at 200 rpm for 24 hr at 60°. Following incubation, the enzyme was inactivated by incubation for 10 min at 100°. The solution was filtered and relative xylulose concentration was determined by HPLC analysis.

### HPLC system

Ethanol production was measured using two Agilent 1200 series HPLC systems, each equipped with a high-performance autosampler that enables analysis in 96-well plate format. An anion exchange Aminex HPX-97H column (Bio-Rad, Hercules, CA), which allows measurement of sugar, glycerol, and ethanol concentrations, was used to measure sugar concentration in the media. A Fast Acid Analysis column was used for measuring ethanol concentration in the media (allows measurement of ethanol concentrations with a retention time of 5 min, shorter than 23 min when using the Aminex column). Both columns were eluted with 5 mM H_2_SO_4_ at 45°. Flow rate for the Aminex column was 0.6 ml/min, flow rate for the Fast Acid Analysis column was 1.0 ml/min.

### Ethanol and biomass measurements

Cells were grown in 96-well plates at 30° in a 96-well plate shaker at 1050 rpm until stationary phase was reached, as determined by preliminary analysis: 24 and 40 hr, and 13 d on media containing glucose, galactose, and xylulose as a carbon source, respectively. Measurements on xylulose were performed after 7 and 10 d of growth as well. Once stationary phase was reached, the cells’ OD was measured using a Tecan Sunrise microplate reader, and samples from each well were filtered and frozen at −80°. Samples were then thawed and placed in the HPLC system for measurement of ethanol concentrations. Ethanol measurements were corrected for ethanol evaporation based on a linear calibration curve.

### Competition assays

Yeast strains that express a fluorescent marker (*e.g.*, GFP) and a nonfluorescent yeast strain were grown separately overnight. The stationary starters were then inoculated together in the same well of a 96-well plate in equal concentrations, and diluted to a final concentration of 1:1024 of the original starters. The plates were incubated at 30° in a 96-well plate shaker at 1050 rpm for a time interval that allowed the cells to reach stationary phase. Following this time interval, the cells were diluted 1:1024 and again incubated for further growth. This dilution process was repeated in 24 hr intervals for media containing glucose and galactose as a carbon source, and in 72 hr intervals for media containing xylulose. Cell frequency was measured by FACS both at the initial inoculation of the cells in the same well and at each dilution point, measuring 30,000 cells per sample. The differences in the strains’ growth rates were derived from the frequencies measured by FACS. The 96-well format allowed high-throughput analysis of hundreds of strains in each experiment.

### Flow cytometry

FACS analysis was done using a BD LSRII system (BD Biosciences). Flow cytometry was conducted with excitation at 488 nm and emission at 525 ± 25 nm for GFP samples.

### RNA extraction and sequencing

A starter of cells was grown overnight on SC medium containing 2% carbon source (glucose, galactose, or xylulose). The cells were then diluted to OD_600nm_ = 0.1 in 15 ml tubes and grown until they reached mid log phase (0.4 < OD_600nm_ < 0.6). Cells were then harvested by 1 min centrifugation at 4000 rpm, the supernatant was discarded, and they were frozen in liquid nitrogen.

RNA was extracted using the Nucleospin 96 RNA kit with modifications for working with yeast. Lysis was performed by mixing the cells with 450 µl lysis buffer [1 M sorbitol (Sigma S1876), 100 mM EDTA 0.5 M, and 100 U/ml lyticase]. The lysis mixture was transferred to a 96-well plate that was incubated at 30° for 30 min. The plate was then centrifuged for 10 min at 2500 rpm, and the supernatant was transferred to a 96-well plate provided by the Nucleospin 96 RNA kit, followed by extraction as described in the kit protocol.

Labeled cDNA was created from RNA extracts, and cDNA was barcoded and then sequenced in the Illumina HiSequation 2500 system, using a Truseq SR Cluster Kit v3 -cBot-HS cluster kit and a Truseq SBS Kit v3-HS run kit (50 cycles).

### Processing and analysis of sequenced RNA

Processing and analysis of sequenced RNA was as described in Voichek *et al.* (2016).

### Data availability

Supplemental Material, Table S1 includes a list of all strains included in our screen, as well as all ethanol, OD, and competition measurements. Gene expression data is available at the SRA database, under BioProject PRJNA381594.

## Results

### Screening metabolic genes for their contribution to xylulose growth and fermentation

We chose 411 metabolic enzymes and regulatory factors associated with central carbon metabolism (Figure 1A), and screened for those that affect growth or fermentation on glucose, galactose, and xylulose (Table S1). This was done by assembling a collection of strains that are deleted or heterozygote for the nonessential or essential genes, respectively.

Temporal analysis of ethanol production and sugar depletion revealed that wild-type cells grown on 2% glucose consumed the sugar within 24 hr, producing 7 g/L of ethanol (Figure 1B and Figure S1 in File S1), which corresponds to ∼70% of the theoretical yield (Zhang *et al.* 2015). Growth on galactose produced a similar amount of ethanol but at a slower rate, depleting the sugar only after ∼40 hr of growth. Growth on 2% xylulose was significantly slower: after 13 d of growth, xylulose could still be detected in the medium. Still, a significant amount of ethanol was produced (4.5 g/L), corresponding to ∼65% of the ethanol yield on glucose.

The mutant strains were similarly characterized, by measuring the OD and the amount of ethanol produced from the three carbon sources. On xylulose, two additional time points were taken to assess ethanol production and OD during midlogarithmic growth as well. We further measured the growth rates of all mutant strains in the three conditions studied using a sensitive competition assay (Figure 1C and Table S1). Mutant strains were co-incubated with GFP-labeled wild-type cells, and their relative abundance was quantified by flow cytometry at stationary phase. The relative growth rate was calculated by measuring these frequencies temporally, following several dilutions of the cultures. 

### Growth on xylulose shows a signature of respiratory metabolism

**Figure 2:**
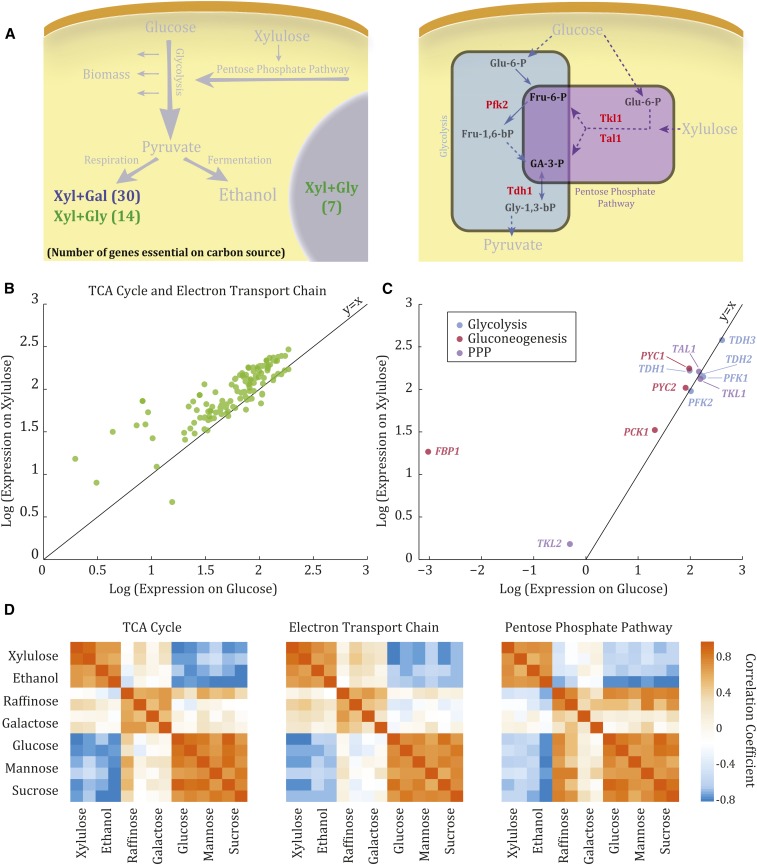
Genes essential for growth on Xyl are mostly required for growth on nonfermentable carbon sources. (A) Genes essential for growth on Xyl are mostly essential for growth on Gal and Gly as well. Left panel: pathways to which the genes essential for growth on Xyl and Gal or Xyl and Gly belong. Indicated in parentheses is the number of genes essential for growth on the carbon sources denoted. Right panel: a scheme of glycolysis and the PPP, in which the four genes required for growth on Xyl specifically are highlighted (red). (B) Growth on Xyl upregulates genes of the TCA cycle and electron transport chain. Shown is log(expression) on Xyl *vs.* log(expression) on Glu of genes of the TCA cycle and ETC. A *t*-test was used to calculate the significance of the shift in the mean of the distribution relative to zero, giving a *p*-value of 1.82 × 10^−6^. (C) Growth on Xyl upregulates gluconeogenic genes. Shown is log(expression) on Xyl *vs.* log(expression) on Glu of genes of glycolysis, gluconeogenesis, and the PPP. (D) The expression pattern of genes of the TCA cycle, ETC, and PPP during growth on Xyl correlates with that of cells growing on ethanol. Expression data for all carbon sources other than Xyl was taken from Gasch *et al.* 2000. Fru-1,6-bP, fructose-1,6-bisphosphate; Fru-6-P, fructose-6-phosphate; Ga-3-P, glyceraldehyde-3-phosphate; Gal, galactose; Glu-6-P, glucose-6-phosphate; Gly, glycerol; Gly-1,3-bP, 1,3-bisphosphoglycerate; PPP, pentose phosphate pathway; TCA, tricarboxylic acid; Xyl, xylulose; Glu, glucose; ETC, electron transport chain.

Of the 411 strains examined, 55 were nonviable (or showed very poor growth) on xylulose. Of these, 30 were nonviable also on galactose. Of the 25 remaining mutants, previous analysis indicated that 21 were nonviable on glycerol, a carbon source that can only be respired but not fermented (McAlister-Henn and Thompson 1987; Dimmer *et al.* 2002; Dudley *et al.* 2005; Merz and Westermann 2009; Na *et al.* 2014) (Table S2). Therefore, only four genes were essential specifically for growth on xylulose: *TKL1*, *TAL1*, *PFK2*, and *TDH1* (Figure 2B).

The majority of the 30 genes that were essential for growth on both galactose and xylulose (but not on glucose) are involved in the electron transport chain or participate in its regulation (Figure 2A, left panel). Similarly, the majority of the 25 strains that were nonviable on both xylulose and glycerol were again associated with the respiratory pathways, including the TCA cycle and the electron transport chain. In addition, several of these genes were part of the Snf1 regulatory network, which is responsible for deactivating the glucose repression response (Schüller 2003). Therefore, despite the considerable ethanol yield during growth on xylulose, growth on this sugar depended on respiratory genes in a manner highly similar to that of growth on carbon sources that are strictly respired, such as glycerol.

To better understand this requirement for respiratory genes, we asked whether a respiratory signature is evident also in the pattern of gene expression. To this end, we measured the genome-wide transcription programs of wild-type cells growing on xylulose and glucose. Consistent with the genetic requirements described above, genes of the TCA cycle, electron transport chain, and gluconeogenesis were highly induced on xylulose compared to glucose (Figure 2, B and C). Further, the expression pattern on xylulose was most similar to that of cells grown on the strictly respiratory carbon ethanol, as evident by comparison of this expression program to that reported on multiple carbon sources (Figure 2D, Gasch *et al.* 2000). In addition to ethanol, these carbon sources also included the fermentative sugars glucose, sucrose, and mannose, and the partially respiratory sugars galactose and raffinose. Taken together, both the genetic requirements and the gene expression signature were indicative of a largely respiratory metabolism of xylulose, despite high ethanol yield.

### Enzymes that link glycolysis to the pentose phosphate pathway are essential for growth on xylulose

As described above, of the 55 genes that were essential for growth on xylulose, four were not essential for growth on either glycerol or galactose: *TKL1*, *TAL1*, *PFK2*, and *TDH1*. As we describe below, these genes are positioned at critical nodes, linking the PPP with glycolysis through intermediates common to both pathways, and may therefore be required to allow the entry of carbon to central metabolism through the PPP.

Two of these genes, *TAL1* and *TKL1*, catalyze PPP reactions required for conversion of xylulose into glycolytic intermediates (Figure 2A, right panel): Tkl1 converts xylulose-5-phosphate and ribose-5-phosphate into sedoheptulose-7-phosphate and glyceraldehyde-3-phosphate (GA-3-P), while Tal1 converts the latter two into erythrose-4-phosphate and fructose-6-phosphate (Fru-6-P) (Figure S2 in File S1). In addition to their role in the PPP, there is evidence indicating that overexpression of these two genes can drive ethanol production in several different growth conditions (Hasunuma *et al.* 2014; Cao *et al.* 2014; Kobayashi *et al.* 2017), although *TAL1* expression was not significantly induced during growth on xylulose compared to glucose (Figure 2C). *TKL1* was slightly downregulated, while its paralog, *TKL2*, was induced (Figure 2C). This induction of the minor transketolase isoform was therefore not able to compensate for the *TKL1* deletion, in agreement with early studies of the two enzymes in which the Δ*tkl1* mutant showed immeasurable transketolase activity (Schaaff-Gerstenschläger *et al.* 1993). In addition, Tkl1 is primarily localized to the nucleus, while Tkl2 is primarily localized to the cytoplasm (Koh *et al.* 2015), suggesting a functional difference between the two, perhaps a role in nucleotide production, for which the PPP is responsible.

The two additional genes that were required specifically for growth on xylulose code for enzymes of the glycolysis pathway: *PFK2* and *TDH1* (Figure 2A, right panel). *TDH1* encodes one of three GA-3-P dehydrogenase isozymes, which catalyze the conversion of GA-3-P to 1,3-bis-phosphoglycerate in glycolysis. *PFK2* encodes the β subunit of phosphofructokinase, another key enzyme of glycolysis that catalyzes the formation of fructose 1,6-bisphosphate from Fru-6-P. These two enzymes link the PPP with glycolysis, allowing xylulose to enter central carbon metabolism, likely justifying their requirement for growth on xylulose (Figure 2A, right panel).

During growth on glucose or galactose, Δ*pfk2* and Δ*tdh1* grew at the same rate as wild-type cells, suggesting that their depletion is efficiently compensated for by their respective isozymes. The strict requirement for *TDH1* on xylulose may be explained by its expression pattern: while *TDH1* was induced on xylulose compared to glucose, its two isozymes, *TDH2* and *TDH3*, were not (Figure 2C). This expression pattern was consistent with that observed under stress or respiratory conditions (Gasch *et al.* 2000; Delgado *et al.* 2001).

In contrast, the expression of *PFK2*, or of its isozyme *PFK1*, was not noticeably different between xylulose and glucose (Figure 2C). However, *FBP1*, which catalyzes the reverse reaction in gluconeogenesis, was induced on xylulose, perhaps explaining why both enzymes were needed to maintain the directionality of the glycolytic flux downstream, toward energy and biomass production. Notably, this induction pattern has also been observed under stress or respiratory conditions (Gasch *et al.* 2000; Delgado *et al.* 2001).

### Exploring genes that alter xylulose fermentation

**Figure 3:**
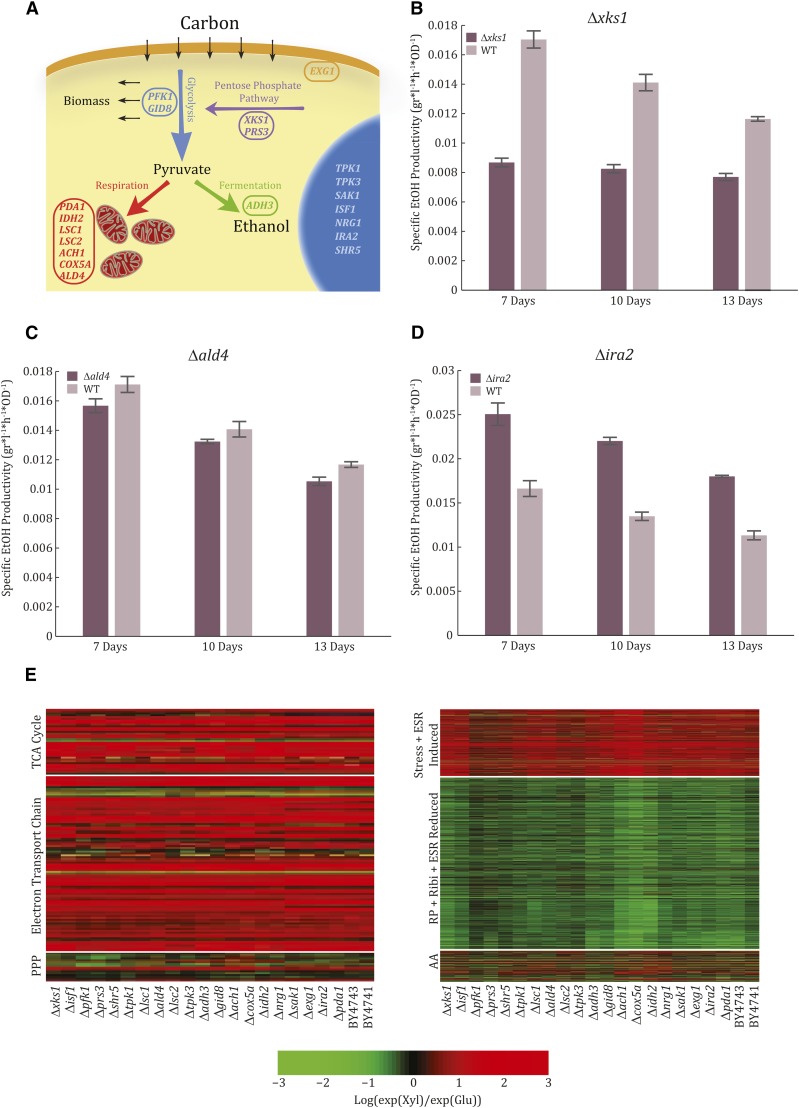
Mutant strains whose ethanol production phenotype differs from the wild-type (WT) show WT-like expression patterns. (A) A schematic overview of the 20 validated candidate genes in the context of the pathways they take part in. (B) An example of a strain that produces less ethanol than the WT at a lower final OD, Δ*xks1*. Shown is the specific ethanol productivity of the mutant (maroon) *vs.* the WT (lavender) reached after 7, 10, and 13 d of growth on xylulose (Xyl). (C) An example of a strain that produces less ethanol than the WT at the same final OD reached by the WT, Δ*ald4*. Shown is the specific ethanol productivity of the mutant (maroon) *vs.* the WT (lavender) reached after 7, 10, and 13 d of growth on Xyl. (D) An example of a strain that produces ethanol amounts similar to those of the WT at a lower final OD, Δ*ira2*. Shown is the specific ethanol productivity of the mutant (maroon) *vs.* the WT (lavender) reached after 7, 10, and 13 d of growth on Xyl. (E) The gene expression of the different mutants resembles that of the WT strain. Shown is the difference (log ratio) between expression on Xyl and expression on glucose (Glu) of genes of the tricarboxylic acid (TCA) cycle, pentose phosphate pathway (PPP), and electron transport chain (left) and amino acid biosynthesis (AA), ribosomal proteins (RP), ribosomal biogenesis (Ribi), environmental stress response (ESR)-reduced, ESR-induced, and stress genes. Data is shown for WT strains as well as all mutants examined. Gene groups as described in Tamari *et al.* (2014).

We next searched for mutants that maintained growth on xylulose, but were perturbed in their fermentation capacity, producing less (or more) ethanol than wild-type cells. Our screen pointed to 39 candidates, for which we generated the mutations anew, and measured their ethanol production, biomass, and growth rate on xylulose. Twenty of these strains, which showed the most consistent results, were kept for further analysis (Figure 3A, Figure S3A in File S1, and Table S3).

We classified the mutants into three groups (Figures S4–S6 in File S1, Table S3, and Table S4). First, we included six strains that produced low amounts of ethanol and reached a low final OD, giving specific ethanol productivity that was lower than wild-type. This phenotype indicated a general deficiency of growth on xylulose. Genes assigned to this group were *XKS1*, *PFK1*, *ISF1*, *SHR5*, *PRS3*, and *TPK1*. Xks1 is the xylulokinase enzyme that converts D-xylulose and ATP to xylulose-5-phosphate and ADP (Richard *et al.* 2000), and it was therefore expected that the ethanol productivity of its heterozygote deletion would be low (Figure 3B). Low ethanol yield was also found for the strain deleted of *PFK1*, the α subunit of phosphofructokinase, whose β subunit was found to be essential for growth on xylulose. Second, we considered strains that produced less ethanol than wild-type cells, while reaching the same final OD. Genes assigned to this group were *TPK3*, *ALD4*, *ACH1*, and *COX5A*. These mutants appeared to divert flux away from fermentation (Figure 3C). The final group of strains produced wild-type-like amounts of ethanol but reached a lower final OD, indicating increased specific ethanol productivity (Figure 3D). Genes assigned to this group included *IRA2*, *NRG1*, *SAK1*, *EXG1*, *IDH2*, *PDA1*, *LSC2*, *GID8*, *LSC1*, and *ADH3*. Notably, none of the selected strains were deficient in growth on glucose or galactose (Figure S3B in File S1). Expression of 14 of these 20 genes was upregulated by xylulose, as compared to glucose (Figure S7 in File S1).

### Growth on xylulose induces respiratory and stress-related genes in mutant strains

As can be seen, the selected mutants were associated with different metabolic functions (Figure 3A and Table S3). Since fermentation yield correlates with a well-defined transcription program, we asked whether the mutants show a consistent expression signature that correlates with their effects. To this end, we compared the gene expression program of each mutant when grown on glucose *vs.* xylulose. All mutants responded to growth on xylulose-containing media in essentially the same way: they induced respiratory genes such as those of the TCA cycle and electron transport chain, upregulated specific isozymes of the PPP, and downregulated ribosomal-associated genes (Figure 3E). Therefore, the mutant phenotypes were not explained by differential regulation of gene expression between glucose and xylulose.

**Figure 4:**
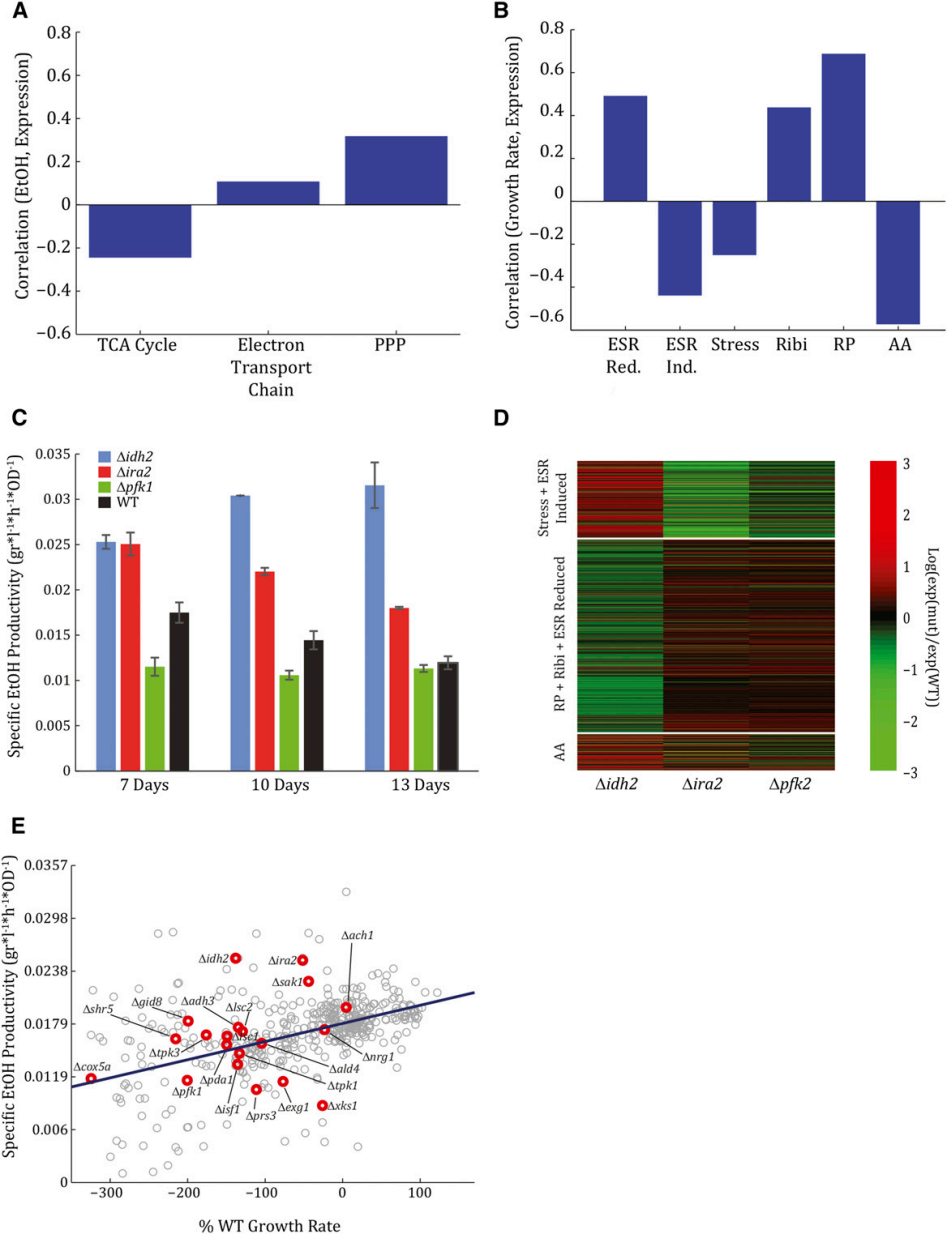
Mutant expression patterns do not correlate with ethanol production, growth rate, or phenotype. (A) Mutant expression patterns do not correlate with ethanol production. Shown is a bar plot of the correlation between mean expression levels of genes belonging to the tricarboxylic acid (TCA) cycle, electron transport chain, and pentose phosphate pathway (PPP) *vs.* the amount of ethanol produced by each deletion strain. Expression is defined as the difference (log ratio) between each deletion strain and the wild-type (WT). (B) Mutant expression patterns do not correlate with growth rate. Shown is a bar plot of the correlation between mean expression levels of genes belonging to the environmental stress response (ESR)-induced, ESR-reduced, amino acid biosynthesis (AA), ribosomal proteins (RP), ribosomal biosynthesis (Ribi), and stress genes *vs.* the strains’ growth rate compared to the WT. Expression is defined as the difference (log ratio) between each deletion strain and the WT. (C) Specific ethanol productivity of Δ*ira2*, Δ*idh2*, and Δ*pfk1* when grown on xylulose over time. Shown is the specific ethanol productivity after 7, 10, and 13 d of growth for each of the mutants as well as the WT. (D) Expression patterns of Δ*ira2*, Δ*idh2*, and Δ*pfk1* during logarithmic growth on xylulose. Difference (log ratio) between expression on xylulose of each deletion strain and the WT, of genes belonging to the AA, RP, Ribi, ESR-reduced, ESR-induced, and stress gene modules. (E) Specific ethanol productivity positively correlates with growth rate. Shown is specific ethanol productivity measured after 7 d of growth *vs.* percent of WT growth rate for all mutants screened. Candidate mutants whose phenotypes were validated are depicted in red.

We next asked whether mutant fermentative capacity correlated with absolute expression levels of respiratory genes on glucose or on xylulose. No correlation was identified when comparing the ethanol yield of the different strains and the associated expression levels of genes in the TCA cycle, electron transport chain, or PPP (Figure 4A). This lack of correlation was consistent with previous results from our lab, comparing xylulose fermentation in different wild-type strains (Tamari *et al.* 2014), as well as with other studies that have shown induction of genes associated with metabolism of nonfermentable carbon sources (Scalcinati *et al.* 2012; Shen *et al.* 2013; Alff-Tuomala *et al.* 2016; Sato *et al.* 2016). Similarly, no correlation was identified between growth rate of the mutants and the expression of growth-associated genes: genes induced or downregulated by the environmental stress response (ESR-induced and ESR-reduced, respectively), stress genes, and ribosomal biogenesis genes (Ribi) (Figure 4B). Consistent with previous results, growth rates were positively correlated with the expression of ribosomal proteins (RP) (*c* = 0.69), and negatively correlated with expression of amino acid biosynthesis genes (AA) (*c* = −0.57). This negative correlation with AA genes was previously described in cells growing on xylulose, as well as deletion mutants and yeast growing on several drugs (Tamari *et al.* 2014). Common to all of these conditions and genetic perturbations is a lack of evolutionary adaptation, which may lead to differential expression patterns of growth-associated genes.

Together, we show that expression patterns of mutants perturbed in their fermentation capacity did not correlate with ethanol production, and that their growth rates were not correlated with growth-associated genes. In addition, respiratory and stress genes were induced, again indicating that ethanol production occurred despite a respiratory gene expression program.

### Strains with similar phenotypes show contrasting expression patterns

The lack of correlation between specific ethanol productivity and the expression of respiratory genes was surprising to us; therefore, we examined more directly whether mutants assigned to the same phenotypic class also showed consistent expression patterns (Figure S8 in File S1). To this end, we systematically screened for differential expression of gene modules, defined by coexpression or cofunctionality (Ihmels *et al.* 2002). However, we could not detect consistent expression changes that distinguished the different phenotypic classes.

As a specific example, consider the mutants Δ*ira2* and Δ*idh2*, both of which showed increased specific ethanol productivity compared to wild-type cells (Figure 4C). Despite showing a similar phenotype, their expression pattern was largely different (Figure 4D): Δ*ira2* downregulated stress genes and induced the RP and Ribi genes. By contrast, Δ*idh2* upregulated stress genes and downregulated RP and Ribi genes. This opposite expression pattern is consistent with gene function: Ira2 is a GTPase-activating protein that is a negative regulator of the Ras pathway. Deletion of *IRA2* therefore results in a constitutively active Ras (Wang *et al.* 2004; Zaman *et al.* 2008) that mimics high-glucose conditions, and has also been shown to improve xylose fermentation (Sato *et al.* 2016). Idh2, on the other hand, is a subunit of the mitochondrial isocitrate dehydrogenase complex that catalyzes the oxidation of isocitrate to α-ketoglutarate in the TCA cycle. Disruption of this complex likely impairs the flux through the TCA cycle, necessitating further induction of respiratory and stress genes.

Our data also included strains whose phenotype was distinct, but showed similar gene expression patterns. An example is Δ*ira2*, described above, and Δ*pfk1*, which showed decreased specific ethanol productivity compared to the wild-type (Figure 4, C and D). Therefore, no correlation could be detected between the gene expression pattern and the ethanol production phenotype in this case.

### Xylulose fermentation: a signature of overflow metabolism

Gene expression patterns therefore showed little correlation with the mutants’ fermentation or growth capacity. This may suggest a more global control of fermentation capacity, perhaps in association with the strains’ growth rate. To examine this, we asked whether the specific ethanol productivity correlated with the strains’ growth rate, as measured in a sensitive competition assay. During growth on xylulose, the specific ethanol productivity of the different strains, as measured after 7 d of growth, was well correlated with their growth rate (Figure 4E, *c* = 0.51). Notably, this correlation was specific to growth on xylulose, but was not observed during growth on glucose or galactose.

Correlation between growth rate and ethanol yield has previously been reported for cells growing in continuous cultures limited for glucose (Kaspar von Meyenburg 1969; Postma *et al.* 1989). As a possible explanation, the model of metabolic overflow was proposed. Within this model, respiration occurs at a maximum rate, with any additional flow exceeding this capacity being directed toward fermentation (Vemuri *et al.* 2007). When interpreted in this context, our data suggests that xylulose influx changes in proportion to cell growth rate, while the flux directed toward respiration remains constant, at its maximal limit. Mutants that reduced growth rate therefore directed a larger proportion of their incoming flux toward respiration, leading to reduced specific ethanol productivity.

## Discussion

In this study, we characterized the growth of budding yeast on xylulose, the only known pentose that these cells can naturally utilize. Xylulose is not widely available in nature, yet its incorporation into the metabolic network is possible as it is a natural intermediate of the PPP. Through the PPP, xylulose is fed into the lower part of glycolysis and from there it can be directed toward fermentation or respiration. Notably, under the conditions of our study, a significant fraction of the xylulose carbon was indeed directed into fermentation, reaching a high yield corresponding to 65% of the ethanol yield on glucose.

Despite this significant ethanol production, growth on xylulose was dependent on genes essential for growth on nonfermentable carbon sources (but not required during growth on glucose or on galactose). Therefore, respiration plays a critical role in xylulose metabolism. This reliance on respiratory genes is reflected in, and perhaps explained by, the gene expression pattern, which again mostly resembled that of cells growing on a purely respiratory carbon source, such as ethanol. Therefore, it appears that cellular regulatory networks controlling gene expression and function are not properly tuned for metabolizing xylulose, consistent with its rarity in nature.

We noted that, in most of our deletion strains, the specific ethanol productivity was proportional to the strain’s growth rate (Figure 4E). We interpreted this observation in the context of the model of metabolic overflow (Vemuri *et al.* 2007), which assumes that flow through respiration is limited, so that any excess of incoming flux beyond this limit is directed toward fermentation. In this context, our findings suggested that in most mutants the flux through glycolysis, upstream of the pyruvate branch point, changed in proportion to cell growth rate, while the respiration threshold remained invariant. Therefore, when a mutant decreased growth rate, it also decreased the flux going through glycolysis, thereby lowering the overflow flux used for ethanol production.

As an exception to this general rule, we noted a group of mutants that increased ethanol production but did not increase growth rate (Figure 4E). Within the overflow model, these mutants were expected to affect not only the flux through upper glycolysis, but also the threshold-flux entering respiration. Indeed, Δ*ira2*, for example, reduced expression of respiratory genes, thereby limiting the carbon flux that is directed towards respiration. By contrast, deletion of genes of the respiratory pathway downstream of the pyruvate branch point, such as Δ*idh2*, may have limited the respiratory flux more directly by limiting the function of the associated pathway.

Remarkably, none of our deletion mutants produced more ethanol than the wild-type. Strains that produced wild-type levels of ethanol at a lower final OD had increased specific ethanol productivity by definition, yet the overall yield did not increase. Further, the overall correlation we observed between the amount of ethanol produced and cell growth rate was specific to growth on xylulose, but was not observed during growth on glucose or galactose. In fact, during growth on these two carbon sources, the strains showed little variability in their ethanol production and the final OD that they reached (Figure 1C).

Studying the growth of mutant strains when provided with xylulose as a sole carbon source has given us novel insight into the processes that occur in the cell when carbon enters central metabolism through the PPP. Our results indicate that this special carbon source can be fermented and yet induces and requires respiratory and gluconeogenic genes. This raises the possibility that decoupling the regulation of respiration and gluconeogenesis may better tune pentose metabolism toward fermentation. However, the cell appears to be highly robust against such perturbations, suggesting that this coupling provided a significant benefit during yeast evolution.

## Supplementary Material

Supplemental material is available online at www.g3journal.org/lookup/suppl/doi:10.1534/g3.117.039610/-/DC1.
