# Supplementary material for: The Genetic Requirements for Pentose Fermentation in Budding Yeast
Source: G3 (Bethesda). 2017 Apr 10;7(6):1743–52. doi: 10.1534/g3.117.039610 (PMC5473754; doi:10.1534/g3.117.039610)
Supplement: Supplementary file 1 [file 1743FileS1.pdf]

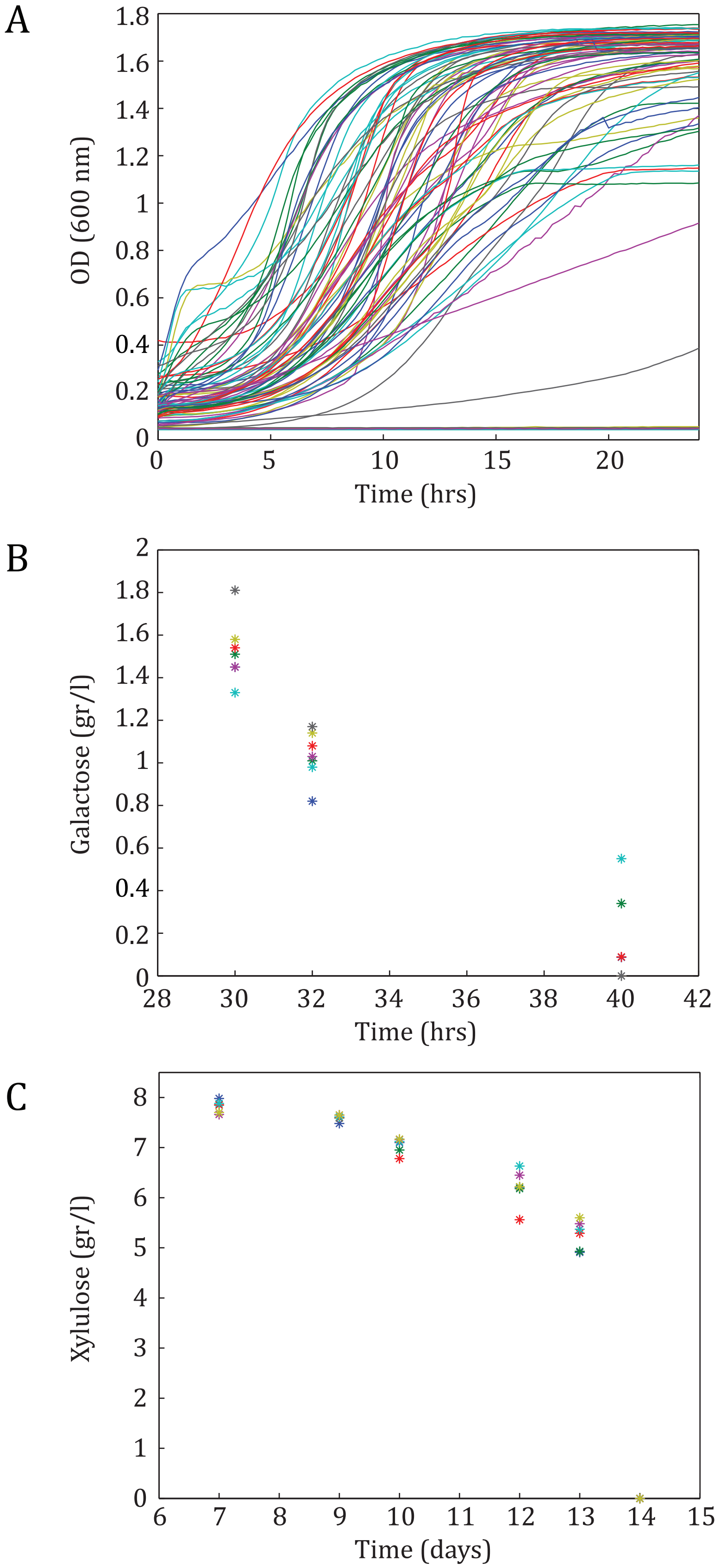

Figure S1 – Sugar consumption of wild type strains on the carbon sources examined

A. Growth curves of one plate of mutants on glucose.

B. Temporal consumption of galactose during growth of BY4741 and BY4743 on SC-galactose.

C. Temporal consumption of xylulose during growth of BY4741 and BY4743 on SC-xylulose. Levels of xylulose after 14 days of growth were below detection level.

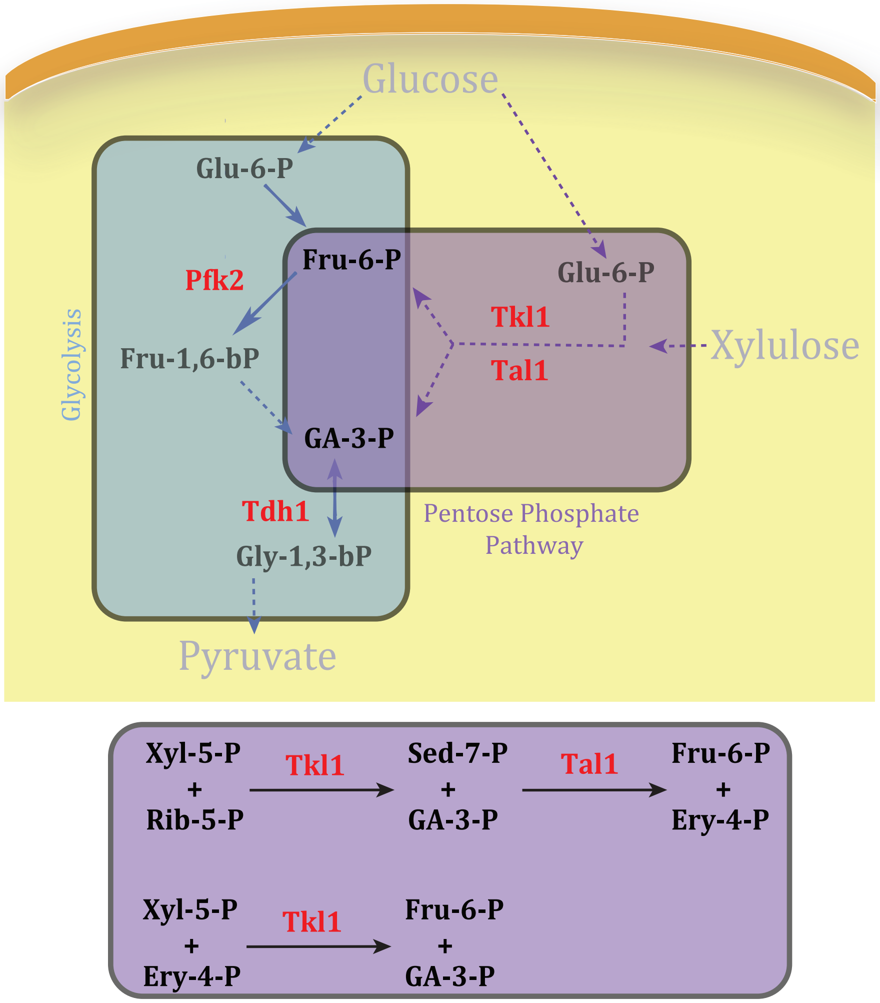

Figure S2 – Transketolase and transaldolase reactions in the PPP
A scheme of the PPP is shown as in Fig. 2A, including the enzymatic reactions of Tkl1 and Tal1.

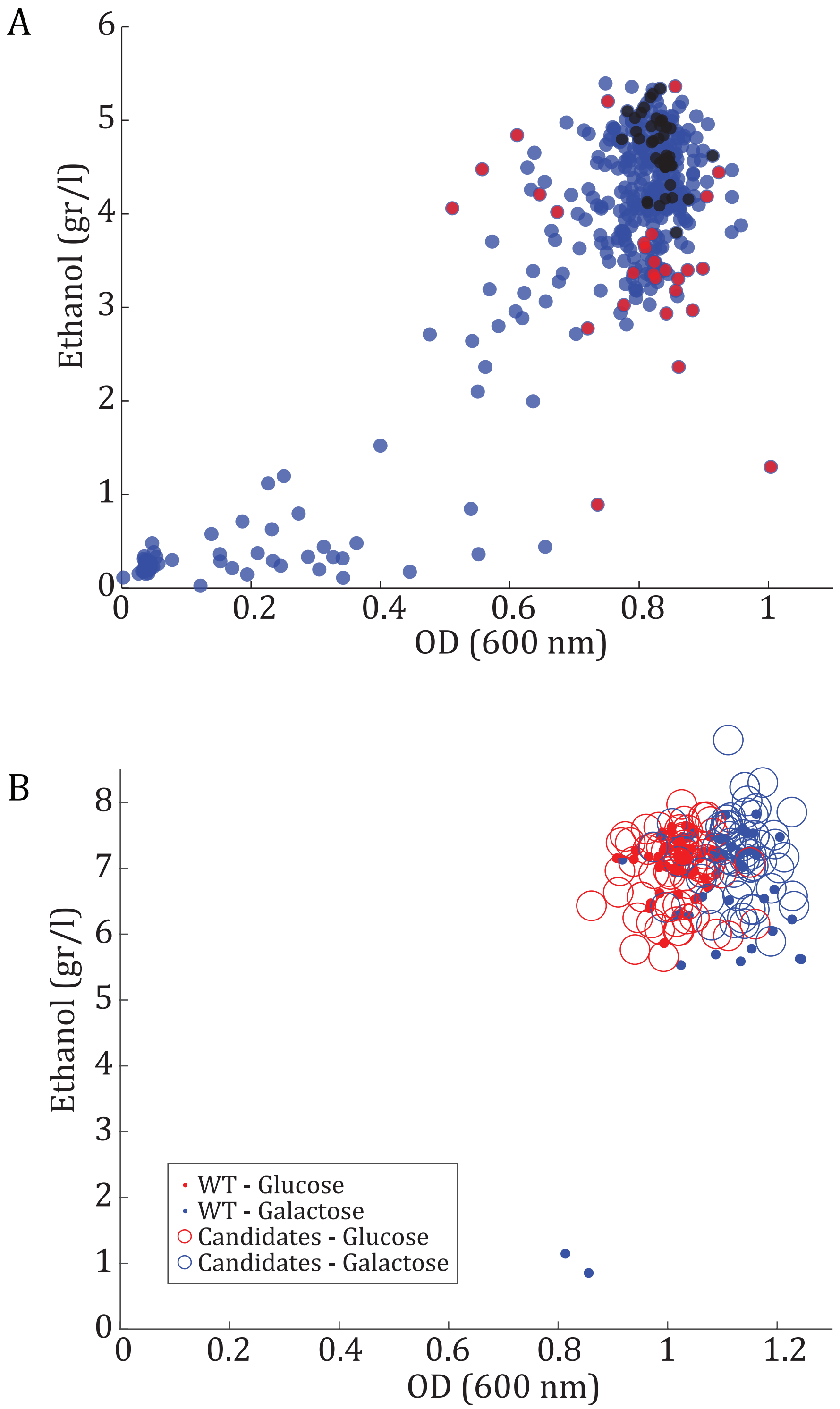

Figure S3 – Ethanol production phenotype of candidate strains varies on xylulose

A. Strains whose ethanol production phenotype diverges from the wild type were taken for further characterization. Shown is the amount of ethanol produced by all of the deletion strains (blue) and wild type repeats (black) after 13 days of growth on xylulose. Shown in red are candidate strains that show an ethanol production phenotype that diverges from the wild type.

B. Ethanol production phenotype of candidate strains is specific to xylulose. Ethanol production vs. final OD produced by wild type and candidate strains on glucose and galactose in the screen.

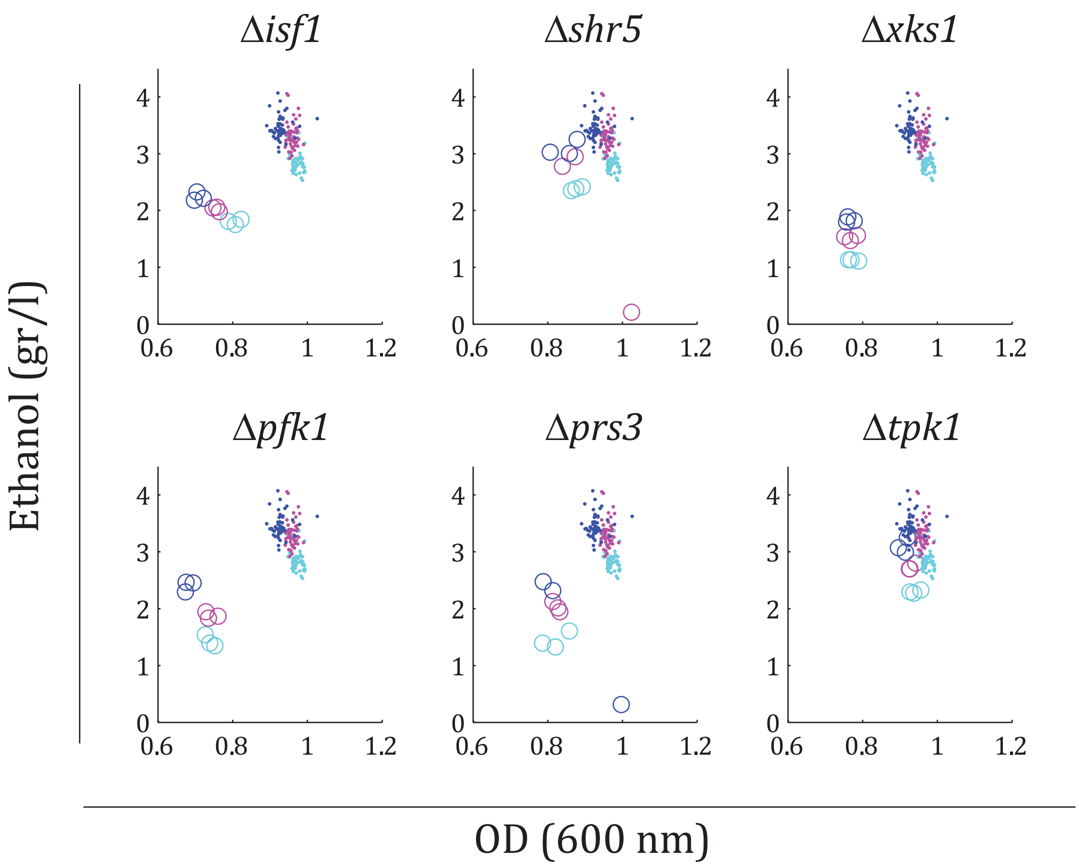

Figure S4 – Ethanol production phenotype of candidate mutants producing low amounts of ethanol at a low final OD

Strains that produce low amounts of ethanol and reach a low final OD. Shown are ethanol vs. OD measurements on SC-xylulose in all three time points (7, 10 and 13 days in cyan, magenta and blue, respectively). Dots show wild type measurements, open circles show the deletion strain measurements.

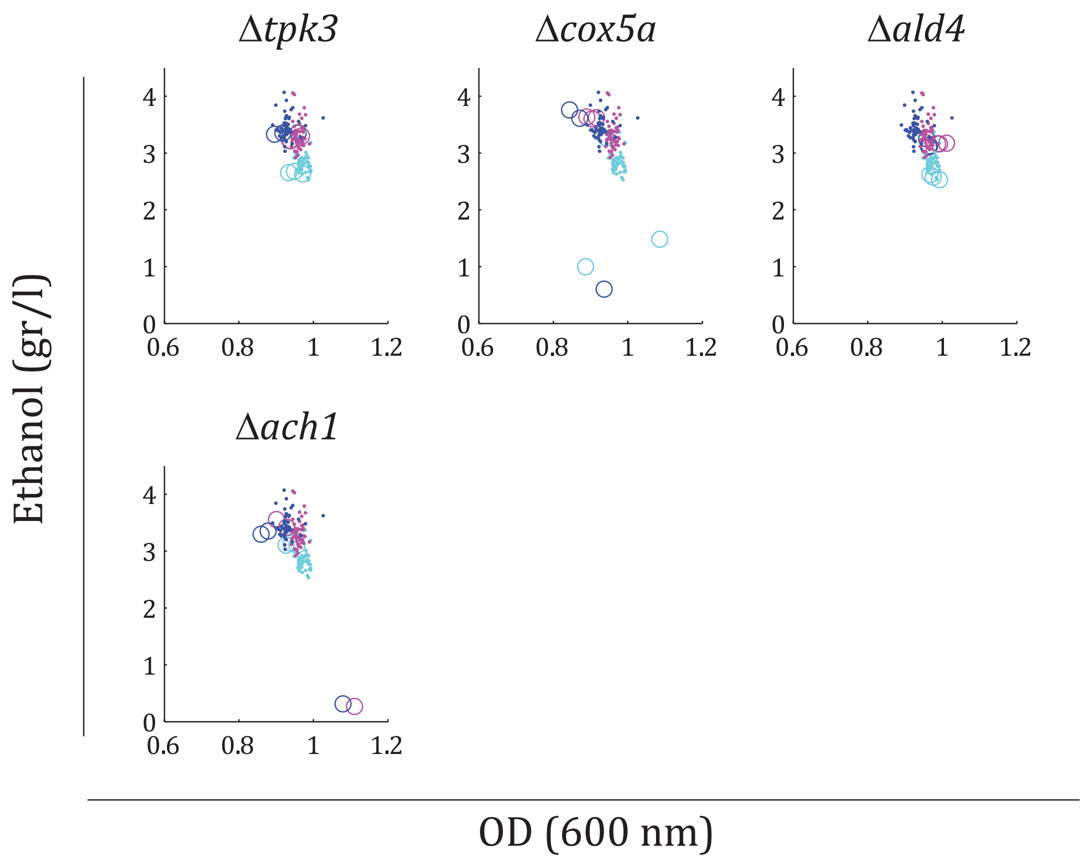

Figure S5 - Ethanol production phenotype of candidate mutants producing low amounts of ethanol at a wild-type final OD

Strains that produce less ethanol than the wild type while reaching a wild type-like final OD. Shown are ethanol vs. OD measurements on SC-xylulose in all three time points (7, 10 and 13 days in cyan, magenta and blue, respectively). Dots show wild type measurements, open circles show the deletion strain measurements.

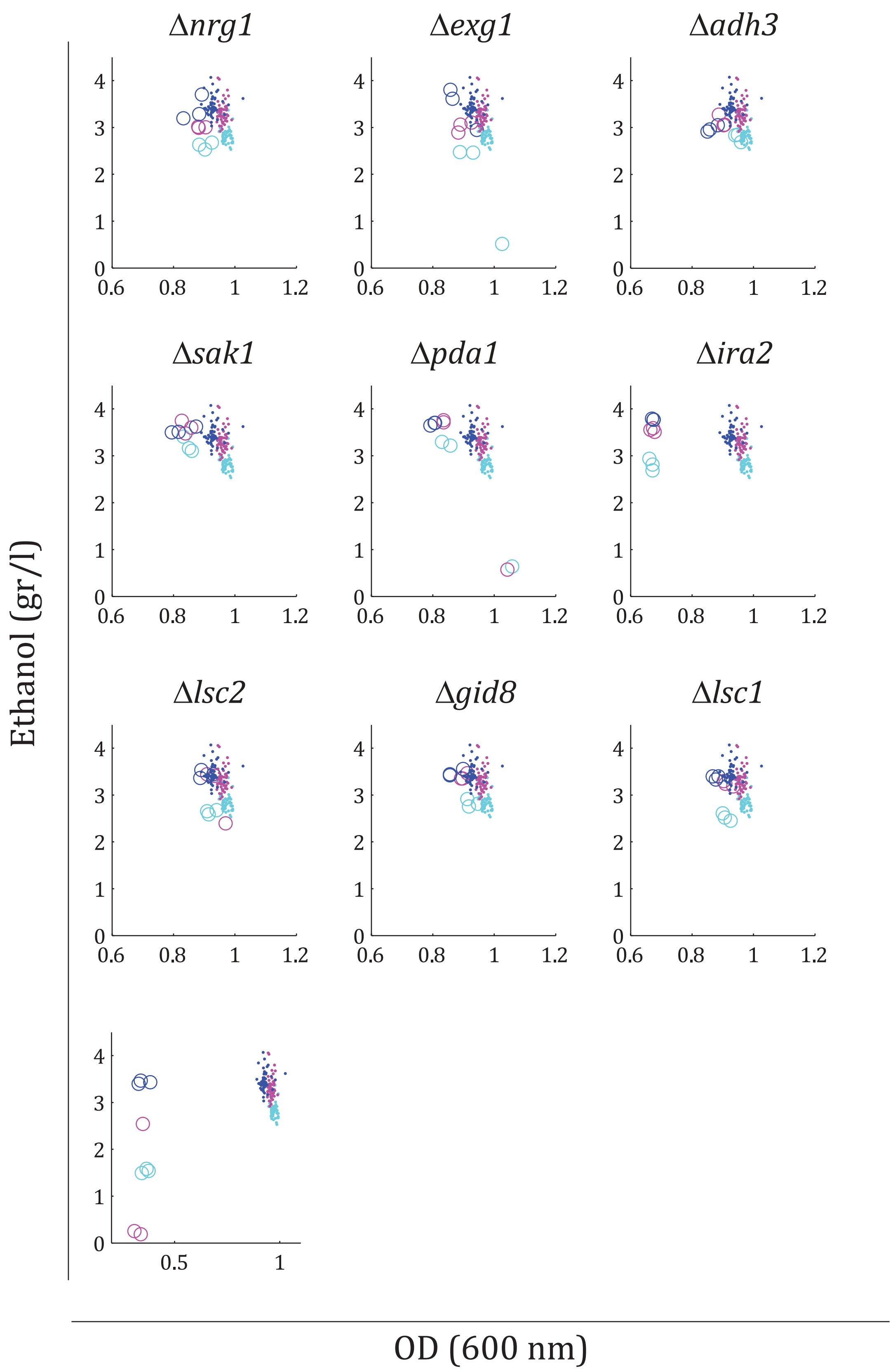

Figure S6 - Ethanol production phenotype of candidate mutants producing wild-type amounts of ethanol at a low final OD

Strains that produce wild type-like amounts of ethanol while reaching a lower final OD. Shown are ethanol vs. OD measurements on SC-xylulose in all three time points (7, 10 and 13 days in cyan, magenta and blue, respectively). Dots show wild type measurements, open circles show the deletion strain measurements.

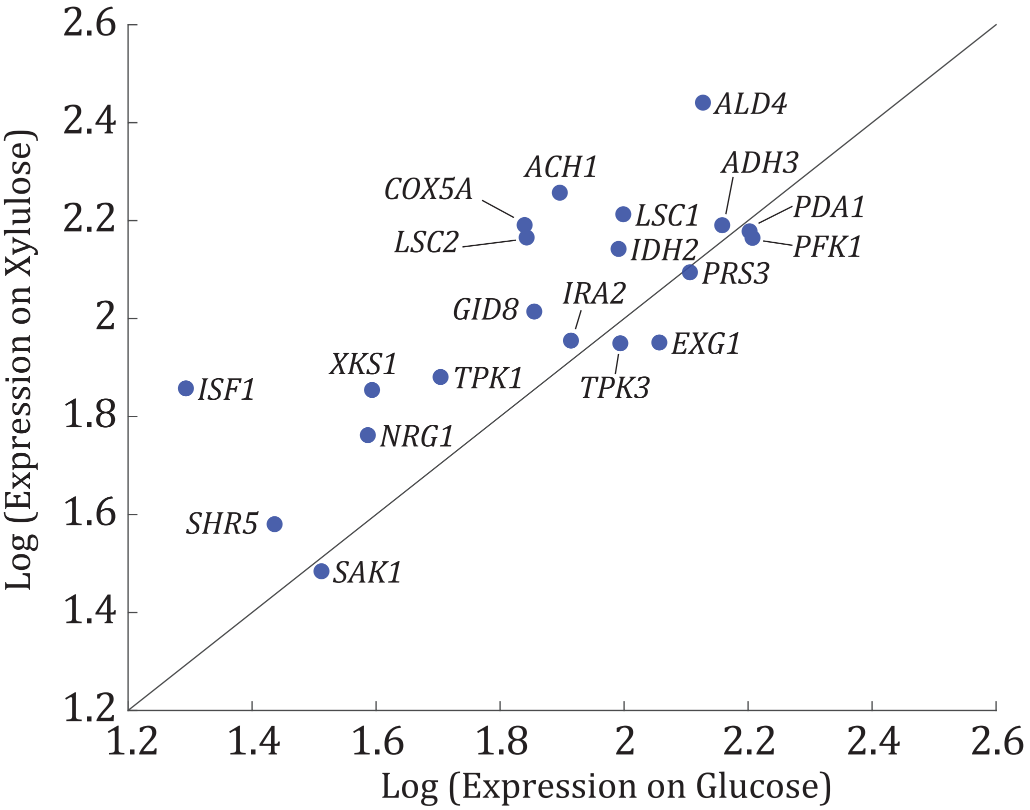

Figure S7 – Expression pattern of candidate genes in the wild-type strain
Log(expression) on xylulose vs. log(expression) on glucose of genes that are deleted in the candidate strains identified in the wild type strain (BY4741).

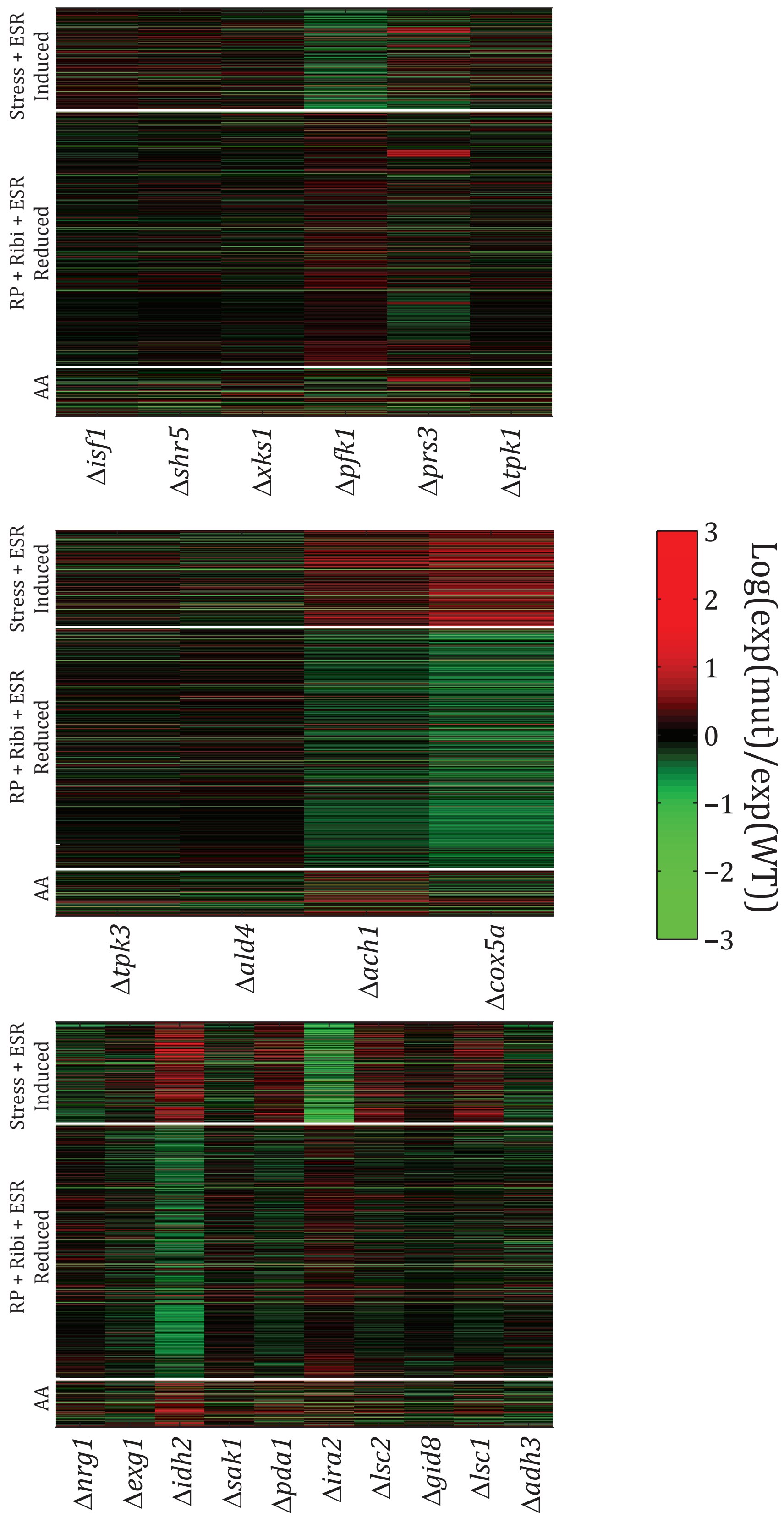

Figure S8 – Expression pattern of growth-associated genes in candidate strains
Difference (log ratio) between expression on xylulose of the deletion strains and expression on xylulose of the wild type, of genes belonging to the stress, amino acid biosynthesis, RP, Ribi, ESR induced and ESR reduced gene modules. Shown are expression levels for each phenotypic group: Strains that produce low amounts of ethanol and reach a low final OD (top); Strains that produce less ethanol than the wild type while reaching a wild type-like final OD (middle); Strains that produce wild type-like amounts of ethanol while reaching a lower final OD (bottom).
